# Factors associated with developmental delay in late preterm infants: the BRISA cohort

**DOI:** 10.1016/j.jped.2025.04.002

**Published:** 2025-05-14

**Authors:** Paulo Ricardo Higassiaraguti Rocha, Gabriela Pap da Silva, Otávio Augusto Gratão, Marco Antonio Barbieri, Viviane Cunha Cardoso, Maria da Conceição Pereira Saraiva, Heloisa Bettiol

**Affiliations:** a Universidade de São Paulo, Faculdade de Medicina de Ribeirão Preto, Departamento de Pediatria, Ribeirão Preto, SP, Brazil; b Universidade de São Paulo, Faculdade de Odontologia de Ribeirão Preto, Departamento de Odontopediatria, Ribeirão Preto, SP, Brazil

**Keywords:** Cohort studies, Risk factors, Premature birth, Cognition, Motor skills

## Abstract

**Objective:**

To investigate the association of sociodemographic characteristics, gestational factors, and birth outcomes with developmental delay from the second year of life in late preterm (LPT) infants.

**Method:**

This study included 327 LPT infants from a cohort started in 2010. Developmental performance was assessed using the Bayley-III screening test. The covariates were obtained with questionnaires and from the maternity records. Hierarchical multiple logistic regression was used for analysis.

**Results:**

Smoking during pregnancy was associated with fine motor and cognitive delays (OR = 2.27, 95 %CI 1.05–4.93 and OR = 2.22, 95 %CI 1.05–4.68, respectively). Living without a partner (OR = 2.98, 95 %CI 1.36–6.52) and intrauterine growth restriction of the child (OR = 2.63, 95 %CI 1.32–5.24) were associated with fine motor delay and neonatal intensive care unit admission with cognitive delay (OR = 2.11, 95 %CI 1.01–4.44).

**Conclusions:**

These factors must be considered when implementing strategies for the diagnosis of possible developmental delays and when designing interventions for LPT children.

## Introduction

Children born at <37 weeks of gestation are classified as preterm. It is estimated that approximately 10 % of children are born prematurely in the world (about 13 million per year) and this condition is a leading cause of death in children under five years of age [1]. Although preterm birth continues to be associated with infant mortality, technological advances and improvements in neonatal care have increased the survival of preterm infants. As a consequence, a large number of studies focusing on the development of preterm infants and on the understanding of the different mechanisms underlying this development have been published in recent years [2, 3, 4, 5, 6].

In general, preterm birth is associated with marked delays in behavioral indicators, particularly during childhood. Compared to their full-term peers, preterm children often perform worse in different developmental assessment tasks such as cognitive and motor tests [7]. These developmental problems tend to be more prominent the lower the gestational age, especially among infants born at <34 weeks of gestation [8].

Studies on the development of preterm infants generally comprise the whole spectrum of prematurity (< 37 weeks of gestation). However, approximately 65 % to 75 % of preterm children are born between 34 and 36 weeks [9]. Although accounting for a high percentage of the preterm population, there is still no consensus in the literature regarding the development of this group [10,11]. As discussed by Ballantyne et al. [2]. and Stene-Larsen et al. [12]. developmental delays in LPT infants can be related to sociodemographic conditions of the family and prenatal and birth risk factors.

Studies investigating behavioral indicators of LPT infants generally compare the performance of this group only to a control group consisting of term children. This approach limits the identification of factors that lead to developmental delays in LPT children and consequently makes it difficult to target intervention strategies in this group [11]. Therefore, the aim of the present study was to investigate the association of sociodemographic factors, maternal lifestyle and reproductive profile, birth attendance, intrauterine growth retardation, and neonatal intensive care unit (NICU) admission with cognitive and motor delays from the second year of life in a sample consisting only of LPT infants.

## Methods

The data used in the present study are part of the project “Etiological factors of preterm birth and consequences of perinatal factors on child health: birth cohorts in two Brazilian cities – BRISA”, (acronym for Brazilian Ribeirão Preto and São Luís Birth Cohort Studies). The BRISA project comprises two cohorts in each city: a prenatal cohort (convenience sample) started during pregnancy, and a birth cohort (population-based sample) followed up since the birth of the child. The present study analyzed the data from the prenatal and birth cohorts of Ribeirão Preto (RP). The first phase of assessments of the RP birth cohort occurred between February 2010 and February 2011. The birth cohort (population-based sample) was conducted between January 1st and December 31st, 2010. The children of the two cohorts were followed up from 2011 to 2013, corresponding to ages 13 to 38 months.

For the prenatal cohort, the pregnant women were recruited in hospitals and health units during a prenatal consultation held up to the 5th month of gestation. Only women with a single pregnancy and an obstetric ultrasound examination performed in the first trimester of gestation were invited to participate in the study. Thus, the RP prenatal cohort included 1400 pregnant women evaluated between 22 and 25 weeks of gestation. For the birth cohort, all parturients from the municipality were invited to participate in the study and 7,752 live births were evaluated, corresponding to 95.7 % of all births during the period. Follow-up occurred during the second and third years of life of the children between 2011 and 2013. All children from the prenatal cohort, including all low birth weight and preterm infants and twins, as well as a sample for comparison of 1.5 children without the characteristics mentioned above, were invited for follow-up, which resulted in 3807 children evaluated in RP. Details of these cohorts, including their follow-ups, have been described previously [13].

For the purpose of the present study, only data from LPT children (single fetuses), who underwent developmental assessment from the second year of life, were included in the analysis, totaling 327 participants.

### Variables

A previously trained team collected the following data within the first 24 hours after delivery using a standardized questionnaire: reproductive health, demographic and socioeconomic data, pregnancy characteristics, and life habits of the pregnant woman. Based on these questionnaires, the following independent variables were obtained for this study: self-reported maternal skin color (white, black, and brown), maternal educational level in years of schooling (≤8 year, 9 to 11 years, and ≥12 years), economic classification according to the Brazilian Economic Classification Criteria of the Brazilian Association of Research Companies[14] (classes A/B, C and D/E, with A/B being the most privileged and D/E the least privileged), marital status (with a partner [married and consensual union] and without a partner), maternal age (<20 years, 20–34 years, and ≥35 years), smoking during pregnancy (yes, if smoking at least one cigarette per day, and no), alcohol consumption during pregnancy (yes, if consuming at least one type of alcoholic beverage during pregnancy, and no), gestational hypertension (yes, reported by the mother, and no), prenatal care (yes and no), type of delivery (vaginal and cesarean), and childbirth care (health insurance/private and public). Regarding NICU admission, the variable was reported by the woman in the questionnaire applied by the team.

Intrauterine growth restriction (IUGR) was defined based on the ratio between birth weight and mean weight for sex and gestational age based on the INTERGROWTH21st curve [15]. A ratio <0.85 was classified as IUGR [16].

Birth between 34^+0^ and 36^+6^ weeks of gestation was considered for the identification of LPT birth. The gestational age was calculated using the date of the last menstruation reported by the mother during the interview and that was determined by ultrasound. In the prenatal cohort, ultrasound was performed during the first phase of the study, between 22 and 25 weeks. In the birth cohort, the earliest ultrasound examination performed by the pregnant woman and recorded in the hospital record or on the prenatal card was defined as the ultrasound-determined gestational age. In the case of compatibility between the ultrasound date and date of last menstruation, assuming an error of ±7 % for ultrasound, the duration of amenorrhea was used for the calculation of gestational age; otherwise, the information provided by ultrasound was considered in both cases [17].

The Bayley Scales of Infant and Toddler Development Third Edition (Bayley-III Screening Test)[18] were used for the assessment of motor and cognitive development. This tool is designed to determine whether development is progressing within expectations or whether further assessment is required. For the evaluation of fine motor development, the instrument is composed of tests aimed at assessing prehension, perceptual-motor integration, and motor planning. Gross motor skills are evaluated by tasks that assess interlimb coordination, displacement, motor planning, and postural stability. Regarding the cognitive subscale, the instrument includes tasks that assess attention, novelty preferences and habituation, problem-solving, exploration and manipulation, concept formation, and other aspects of cognitive development.

For application of the Bayley-III test, the age of LPT children was corrected by subtracting the number of weeks until 40 weeks of gestational age from the chronological age at follow-up. The classification of subscale performance according to the age-related cut scores, established by the scale itself as competent, emerging, and at risk, was considered for analysis. In the present study, subscale performance was dichotomized into competent and emerging/at risk.

### Statistical analysis

The variables were compared between participants (LPT infants) and non-participants in the follow-up using the chi-squared test. Logistic regression was applied to calculate the odds ratio (OR) between the independent variables and the dependent variable. The model was adjusted using a hierarchical approach. The most distal level (sociodemographic) included skin color and educational level of the mother and economic classification. The second level (lifestyle and reproductive profile) included the variables marital status, maternal age, smoking, alcohol consumption, and gestational hypertension. The third level was composed of prenatal care, type of delivery, and childbirth care. The fourth level included IUGR and the fifth level, the most proximal to the outcome, NICU admission. Multiple logistic regression analysis was first performed using the variables of the most distal level to the outcome (sociodemographic). From that level, the variables with *p* < 0.20 were entered into the set of variables of the next level; this process was repeated until the last level. The level of significance was set at <0.05 in all analyses.

All procedures of this study were approved by the Ethics Committee of the University Hospital of the Ribeirão Preto Medical School, University of São Paulo (Approval No 11157/2008). Participation in the present study only occurred after the mothers had properly understood and signed the informed consent form.

## Results

Comparison of the baseline characteristics of participants and non-participants in the follow-up from the second year of life showed differences only for maternal educational level, economic class, and IUGR. There was a lower percentage of mothers with <8 years of schooling (34.2 % vs. 19.1 %) and of economic class D/E (15.2 % vs.7.0 %) in the follow-up, as well as a higher percentage of infants with IUGR (10.9 % vs. 17.4 %) (Table 1).

**Table 1:** Comparison of the characteristics of participants and non-participants in the follow-up from the second year of life. Ribeirão Preto, 2010/13.

Variable		Non-participants	Participants	*p*^a^
		(*n* = 385)	(*n* = 327)	
		*n* (%)	*n* (%)	
**Skin color**				0.05
White		209 (55.1)	208 (64.2)	
Black		44 (11.6)	29 (8.9)	
Brown		126 (33.3)	87 (26.9)	
**Maternal education (years of schooling)**				<0.001
≥12		89 (23.2)	64 (19.7)	
9–11		163 (42.6)	199 (61.2)	
≤8		131 (34.2)	62 (19.1)	
**Economic classification**				0.001
A/B		168 (48.1)	142 (45.1)	
C		128 (36.7)	151 (47.9)	
D/E		53 (15.2)	22 (7.0)	
**Maternal marital status**				0.829
With a partner		333 (86.5)	281 (85.9)	
Without a partner		52 (13.5)	46 (14.1)	
**Maternal age (years)**				0.978
20–34		284 (73.8)	239 (73.1)	
< 20		49 (12.7)	43 (13.1)	
≥ 35		52 (13.5)	45 (13.8)	
**Smoking during pregnancy**				0.076
No		309 (80.3)	279 (85.3)	
Yes		76 (19.7)	48 (14.7)	
**Alcohol consumption**				0.129
No		280 (72.7)	254 (77.7)	
Yes		105 (27.3)	73 (22.3)	
**Gestational hypertension**				0.319
No		302 (78.2)	266 (81.8)	
Yes		81 (21.1)	59 (18.2)	
**Prenatal care**				0.08
Yes		363 (94.3)	317 (96.9)	
No		22 (5.7)	10 (3.1)	
**Type of delivery**				0.702
Vaginal		150 (39.0)	132 (40.4)	
Cesarean		235 (61.0)	195 (59.6)	
**Childbirth care**				0.804
Health insurance/private		189 (49.2)	164 (50.2)	
Public		195 (50.8)	163 (49.8)	
**IUGR**				0.012
No		343 (89.1)	270 (82.6)	
Yes		42 (10.9)	57 (17.4)	
**NICU**				0.898
No		318 (84.6)	276 (84.9)	
Yes		58 (15.4)	49 (15.1)	
**Age at follow-up (months)**				
Mean (SD)			20.15 (4.3)	

Differences in the total number in relation to the reference *n* are due to missing information.

IUGR, intrauterine growth restriction; NICU, neonatal intensive care unit; SD, standard deviation.

a Chi-squared test.

**Figure 1:**
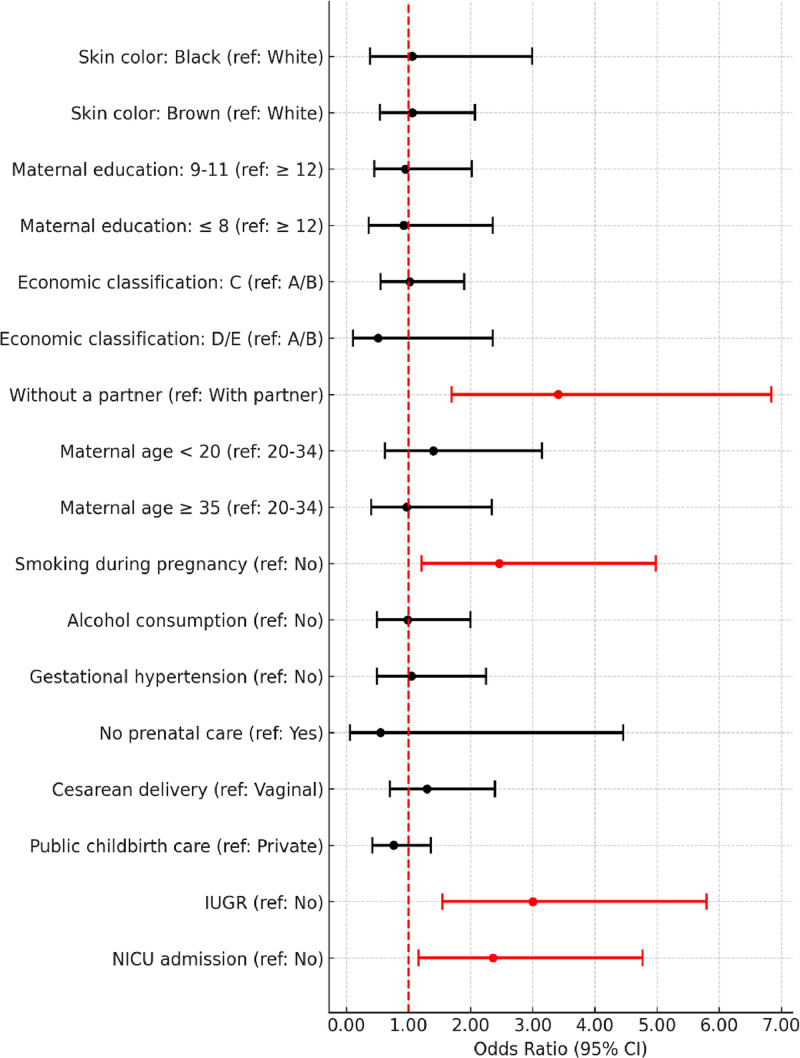
Unadjusted logistic regression analysis of the association of covariates with the classification of developmental delays in the fine motor subscale (Bayley-III). IUGR, intrauterine growth restriction; NICU, neonatal intensive care unit.

**Figure 2:**
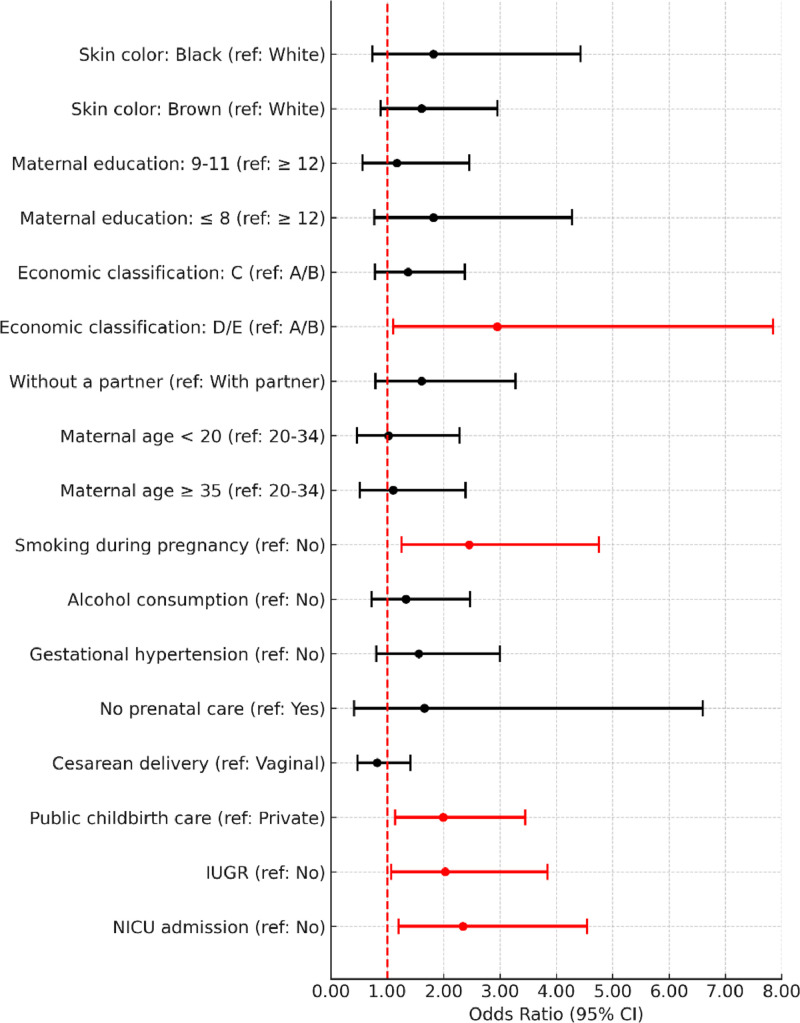
Unadjusted logistic regression analysis of the association of covariates with the classification of developmental delays in the cognitive subscale (Bayley-III). IUGR, intrauterine growth restriction; NICU, neonatal intensive care unit.

In the unadjusted analysis, living without a partner, smoking during pregnancy, IUGR, and NICU admission were associated with fine motor developmental delays (Figure 1). Regarding gross motor developmental delays, only IUGR was associated. The cognitive assessment showed an association between economic class D/E, smoking during pregnancy, public hospital birth, IUGR, and NICU admission with developmental delays (Figure 2).

**Figure 3:**
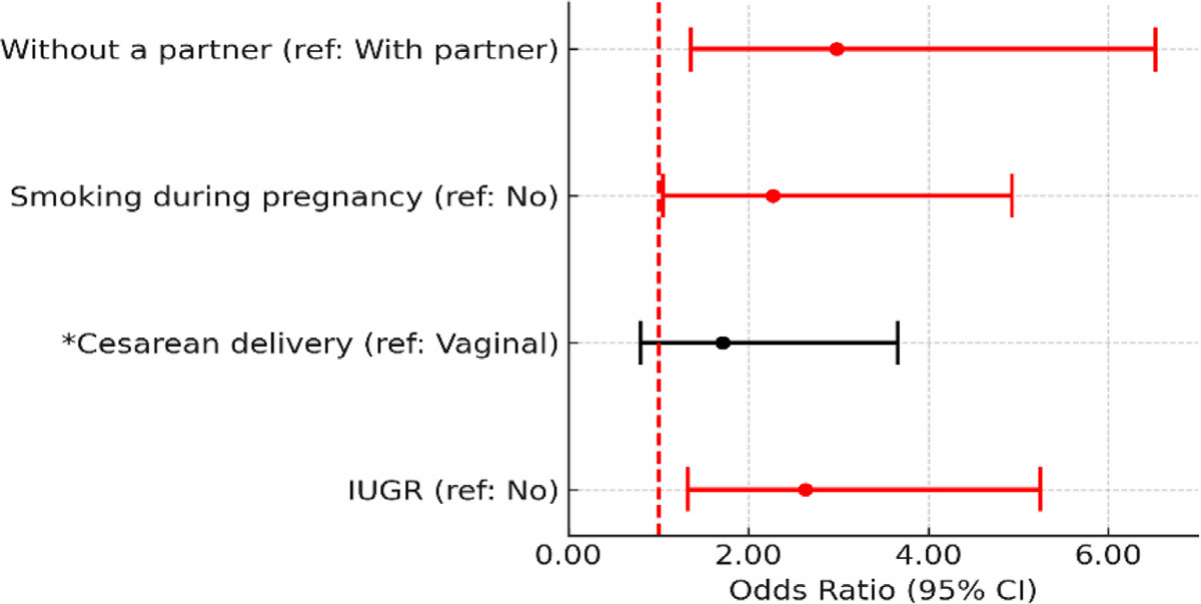
Adjusted logistic regression analysis of the association of covariates with the classification of developmental delays in the fine motor subscale (Bayley-III). *p < 0.20: added to the set of variables of the next level. IUGR, intrauterine growth restriction.

**Figure 4:**
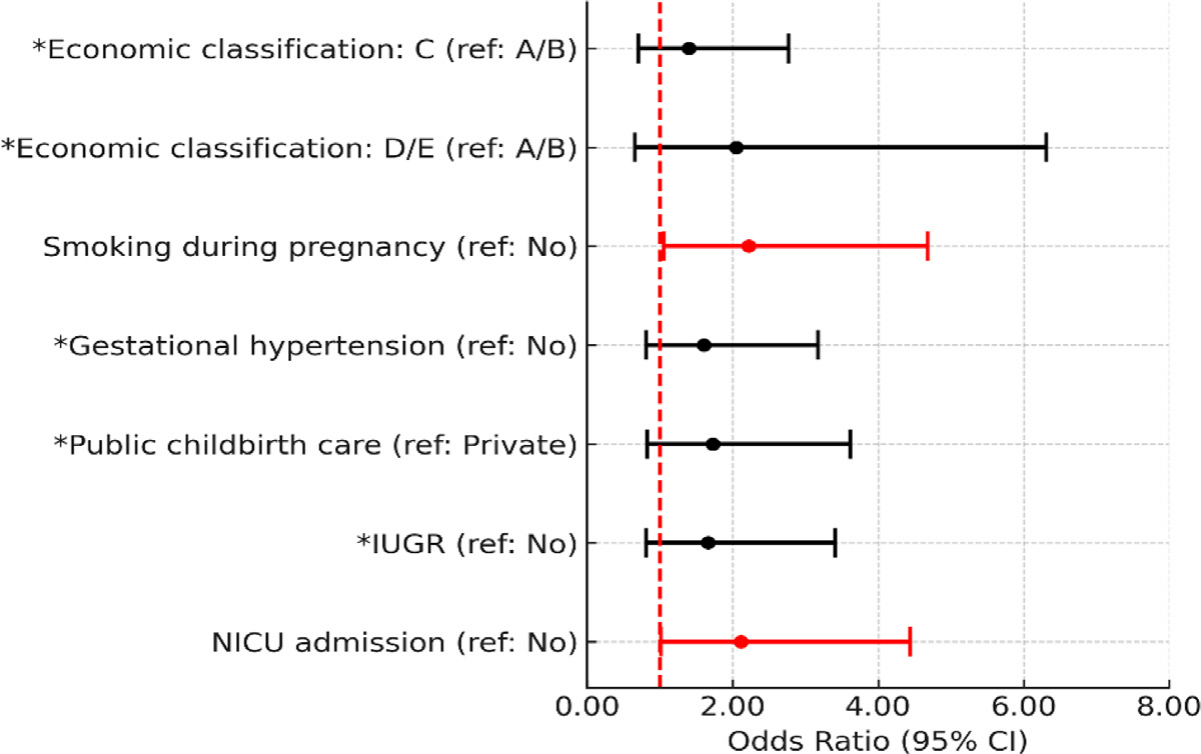
Adjusted logistic regression analysis of the association of covariates with the classification of developmental delays in the cognitive subscale (Bayley-III). *p < 0.20: added to the set of variables of the next level. IUGR, intrauterine growth restriction; NICU, neonatal intensive care unit.

Adjusted analysis showed that the odds of fine motor delays in childhood were approximately three times higher for children born to mothers without a partner (OR = 2.98, 95 % CI 1.36–6.52) and more than two times higher for children born to mothers who smoked during pregnancy and children with IUGR (OR = 2.27, 95 % CI 1.05–4.93 and OR = 2.63, 95 % CI 1.32–5.24, respectively) (Figure 3). None of the variables remained associated with impaired gross motor development. Regarding cognitive development, smoking during pregnancy (OR = 2.22, 95 % CI 1.05–4.68) and NICU admission (OR = 2.11, 95 % CI 1.01–4.44) were associated with cognitive delays (Figure 4). Complete data are available in the Supplementary Material.

## Discussion

The present results revealed that the mother’s marital status, smoking during pregnancy, IUGR and NICU admission are associated with developmental delays in LPT children. In addition, the factors associated with delays are related to the developmental subscale investigated.

The LPT group had greater odds of fine motor developmental delays when the mother did not have a partner. Other studies have also reported perinatal adversities and developmental delays in children born to single mothers [19]. Kim et al. [19]. found a 4-fold higher prevalence of depression among single mothers compared to the control group consisting of mothers with a partner. Some characteristics that are observed more frequently in mothers without a partner, such as lower income, residential instability, a high level of stress, and higher alcohol consumption, were determinants for the association with depression. Depression during pregnancy, in turn, is associated with low birth weight and prematurity [20], conditions commonly reported as predictors of developmental delays.

Smoking during pregnancy was associated with fine motor and cognitive delays in LPT infants. The numerous toxins present in cigarettes can alter placental functions, increasing the risk of perinatal adversities [21]. For example, in the central nervous system, nicotine acts as a neurological teratogen when crossing the placental barrier, triggering nicotine acetylcholine receptors and thus altering the development of nerve tissues. Consequently, intrauterine exposure to nicotine can lead to a decrease in the number of neurons and can cause important changes in sensory-cognitive functions, which could explain in part the difficulties in the execution of cognitive tasks found in children whose mothers smoked during pregnancy [22,23].

Late preterm children born with IUGR exhibited fine motor developmental delays. Other studies have also reported motor alterations in preterm children with IUGR [24,25]. Studies using imaging techniques for morphological and neurophysiological analysis have identified important structural alterations in the nervous system of preterm children born with IUGR that were associated with developmental delays [26,27]. Nevertheless, it should be noted that most of these studies evaluated children born before 34 weeks and the participants were often identified and recruited based on NICU records, which was not the case in the present study.

Admission to the NICU was associated with cognitive developmental delays. Similar results have been reported by Ballantyne et al. [2]. who observed that developmental deficits are greater in LPT children with poor birth outcomes who need to be admitted to the NICU. In general, known factors related to developmental delays such as low birth weight, a low Apgar score, and congenital malformation [28] may also be associated with NICU admission, a fact that would explain in part the results of the present study. Furthermore, it is known that stressful events experienced by newborns, such as frequently undergoing medical interventions at the beginning of life, can alter the organization of the central nervous system and can cause important physiological and behavioral changes over time [29].

None of the variables was associated with gross motor developmental delay. This finding might be explained by the greater variability observed in the development of preterm children [3], which can be attributed to accelerated gains in physical indicators that act as regulators of new behaviors. In other words, the particularity of the preterm growth pattern is an individual restriction that leads to greater variability and oscillations between behavioral states. As a result, despite the delay in the first months of life, preterm children tend to show accelerated gains in gross motor skills at the end of the first year.^4^ Considering this non-linear pattern, it is essential to conduct long-term follow-up for this group, as studies have reported increasing disparities in motor competence between children born preterm and those born at term at the start of the school age, due to the new motor and cognitive challenges characteristic of this stage [3,5].

Some limitations of the present study need to be considered, including the differences in educational level and economic class between non-participants and participants. However, difficulties in following up with sociodemographically vulnerable participants have been a constant challenge in longitudinal cohort studies. Within this context, it is important to use new strategies that increase the retention of these groups in future follow-ups [30]. In the Brazilian context, implementing active follow-up at shorter intervals between assessments, along with greater flexibility in evaluation methods, such as offering home visits or remote interviews, may enhance participants’ engagement with the study and reduce potential barriers related to travel to the assessment site. Another limitation is the use of self-reported information to obtain sociodemographic and gestational covariates, which may have caused information bias. However, these data were collected in the first 24 hours after delivery by a field team that was duly trained by the project coordinators.

As strengths, the authors highlight the cohort design of the study that recruited LPT children from the general population. In addition, to our knowledge, this is the first study that analyzed factors associated with developmental delays in a sample consisting only of LPT infants using a hierarchical approach.

In conclusion, the present study showed that the marital status of the mother, smoking during pregnancy, IUGR, and NICU admission are associated with developmental delays in LPT children. Thus, these factors must be considered when implementing strategies for the diagnosis of possible developmental delays and when designing interventions for LPT children.

## Authors contributions

The conception and design of the study and analysis and interpretation of data: Rocha PRH, Silva GP and Gratão OA. Revising it critically for important intellectual contente: Barbieri MA, Cardoso VX, Saraiva MCA and Bettiol H.

## Conflicts of interest

The authors declare no conflicts of interest.

## Appendix Supplementary materials

Image, application 1

## Footnotes

Supplementary material associated with this article can be found, in the online version, at doi:10.1016/j.jped.2025.04.002.
