# Supplementary material for: Factors associated with developmental delay in late preterm infants: the BRISA cohort
Source: J Pediatr (Rio J). 2025 May 14;101(4):657–64. doi: 10.1016/j.jped.2025.04.002 (PMC12276604; doi:10.1016/j.jped.2025.04.002)
Supplement: Supplementary file 1 [file mmc1.docx]

**JPED-D-24-00601_Supplementary material**

| **Supplementary material.** Unadjusted and adjusted logistic regression analysis of the association of covariates with the classification of developmental delays in the Bayley-III subscales. | | | | | | | |
| --- | --- | --- | --- | --- | --- | --- | --- |
|  | **Bayley-III** | | | | | |  |
|  | **Fine motor** | | | **Gross motor** | | **Cognitive** | |
| **Variable** | **OR (95%CI)** | | **OR* (95%CI)** | **OR (95%CI)** | **OR* (95%CI)** | **OR (95%CI)** | **OR* (95%CI)** |
| **Level 1 - Sociodemographic** |  | |  |  |  |  |  |
| **Skin color** |  | |  |  |  |  |  |
| White | 1 | |  | 1 |  | 1 |  |
| Black | 1.06 (0.38-2.99) | |  | 0.55 (0.35-1.92) |  | 1.82 (0.74-4.43) |  |
| Brown | 1.06 (0.54-2.07) | |  | 1.07 (0.56-2.06) |  | 1.61 (0.88-2.95) |  |
| **Maternal education (years of schooling)** |  | |  |  |  |  |  |
| ≥ 12 | 1 | |  | 1 | *1 | 1 |  |
| 9-11 | 0.95 (0.45-2.02) | |  | 1.12 (0.50-2.51) | 0.99 (0.41-2.36) | 1.17 (0.56-2.45) |  |
| ≤ 8 | 0.92 (0.36- 2.36) | |  | 1.95 (0.78-4.86) | 2.20 (0.77 -6.31) | 1.82 (0.77-4.28) |  |
| **Economic classification** |  | |  |  |  |  |  |
| A/B | 1 | |  | 1 |  | 1 | *1 |
| C | 1.02 (0.55-1.90) | |  | 1.03 (0.54-1.93) |  | 1.37 (0.78-2.38) | 1.40 (0.71-2.77) |
| D/E | 0.51 (0.11-2.36) | |  | 2.04 (0.72-5.80) |  | **2.95 (1.11-7.85)** | 2.05 (0.66-6.31) |
| **Level 2 - Lifestyle and reproductive profile** | |  | | | | |  |
| **Maternal marital status** |  | |  |  |  |  |  |
| With a partner | **1** | | **1** | 1 | *1 | 1 |  |
| Without a partner | **3.41 (1.69-6.84)** | | **2.98 (1.36-6.52)** | 1.69 (0.79-3.58) | 2.04 (0.85-4.87) | 1.61 (0.79-3.28) |  |
| **Maternal age (years)** |  | |  |  |  |  |  |
| 20-34 | 1 | |  | 1 |  | 1 |  |
| < 20 | 1.40 (0.62-3.15) | |  | 0.80 (0.31-2.03) |  | 1.02 (0.46-2.28) |  |
| ≥ 35 | 0.97 (0.40-2.34) | |  | 1.24 (0.55-2.78) |  | 1.10 (0.51-2.39) |  |
| **Smoking during pregnancy** |  | |  |  |  |  |  |
| No | **1** | | **1** | 1 |  | 1 | **1** |
| Yes | **2.46 (1.21-4.98)** | | **2.27 (1.05-4.93)** | 1.16 (0.53-2.57) |  | **2.45 (1.26-4.76)** | **2.22 (1.05-4.68)** |
| **Alcohol consumption** |  | |  |  |  |  |  |
| No | 1 | |  | 1 |  | 1 |  |
| Yes | 0.99 (0.49-2.00) | |  | 1.23 (0.62-2.41) |  | 1.33 (0.72-2.47) |  |
| **Gestational hypertension** |  | |  |  |  |  |  |
| No | 1 | |  | 1 |  | 1 | *1 |
| Yes | 1.05 (0.49-2.25) | |  | 1.18 (0.57-2.47) |  | 1.56 (0.81-3.00) | 1.60 (0.81-3.17) |
| **Level 3 – Antenatal and delivery care** | |  | | | | |  |
| **Prenatal are** |  | |  |  |  |  |  |
| Yes | 1 | |  | 1 |  | 1 |  |
| No | 0.55 (0.06-4.46) | |  | 1.24 (0.25- 6.02) |  | 1.66 (0.41-6.60) |  |
| **Type of delivery** |  | |  |  |  |  |  |
| Vaginal | 1 | | *1 | 1 |  | 1 |  |
| Cesarean | 1.30 (0.70-2.39) | | 1.71 (0.80-3.66) | 1.11 (0.61-2.02) |  | 0.82 (0.47-1.41) |  |
| **Childbirth care** |  | |  |  |  |  |  |
| Health insurance/private | 1 | |  | 1 |  | 1 | *1 |
| Public | 0.76 (0.42-1.36) | |  | 1.14 (0.64-2.05) |  | **1.99 (1.14-3.45)** | 1.73 (0.82-3.62) |
| **Level 4 – Newborn characteristics** | |  | | | | |  |
| **IUGR** |  | |  |  |  |  |  |
| No | **1** | | **1** | **1** | *1 | **1** | *1 |
| Yes | **3.00 (1.55-5.80)** | | **2.63 (1.32-5.24)** | **2.05 (1.04-4.04)** | 1.96 (0.98-3.90) | **2.03 (1.07-3.84)** | 1.66 (0.81-3.41) |
| **Level 5 - Admission** |  |  |  |  |  |  |  |
| **NICU admission** |  | |  |  |  |  |  |
| No | **1** | |  | 1 |  | **1** | **1** |
| Yes | **2.36 (1.16-4.77)** | |  | 1.75 (0.84-3.63) |  | **2.34 (1.20-4.54)** | **2.11 (1.01-4.44)** |
| * p < 0.20: added to the set of variables of the next level.  IUGR, intrauterine growth restriction; NICU, neonatal intensive care unit. | | | | | | |  |
|  | | | | | | |  |
